# Inhibition of 11β-HSD1 Ameliorates Cognition and Molecular Detrimental Changes after Chronic Mild Stress in SAMP8 Mice

**DOI:** 10.3390/ph14101040

**Published:** 2021-10-13

**Authors:** Dolors Puigoriol-Illamola, Júlia Companys-Alemany, Kris McGuire, Natalie Z. M. Homer, Rosana Leiva, Santiago Vázquez, Damian J. Mole, Christian Griñán-Ferré, Mercè Pallàs

**Affiliations:** 1 Pharmacology Section, Department of Pharmacology, Toxicology and Therapeutic Chemistry, Faculty of Pharmacy and Food Sciences, University of Barcelona, Av. Joan XXIII, 27-31, 08028 Barcelona, Spain; dpuigoriol@ub.edu (D.P.-I.); juliacompanysalemany@gmail.com (J.C.-A.); christian.grinan@ub.edu (C.G.-F.); 2 Institute of Neuroscience, University of Barcelona (NeuroUB), Passeig Vall d’Hebron 171, 08028 Barcelona, Spain; 3 MRC Centre for Inflammation Research, Queen’s Medical Research Institute, University of Edinburgh, Edinburgh EH16 4TJ, UK; kris.mcguire@ed.ac.uk (K.M.); damian.mole@ed.ac.uk (D.J.M.); 4 Mass Spectrometry Core, Edinburgh Clinical Research Facility, Queen’s Medical Research Institute, Edinburgh EH16 4TJ, UK; n.z.m.homer@ed.ac.uk; 5 Medicinal Chemistry Section, Department of Pharmacology, Toxicology and Therapeutic Chemistry, Faculty of Pharmacy and Food Sciences, University of Barcelona, Av. Joan XXIII, 27-31, 08028 Barcelona, Spain; rosana.leiva58@gmail.com (R.L.); svazquez@ub.edu (S.V.)

**Keywords:** glucocorticoids, stress, target engagement, aging, epigenetics, oxidative stress, inflammation, neurodegeneration, cognition

## Abstract

Impaired glucocorticoid (GC) signaling is a significant factor in aging, stress, and neurodegenerative diseases such as Alzheimer’s disease. Therefore, the study of GC-mediated stress responses to chronic moderately stressful situations, which occur in daily life, is of huge interest for the design of pharmacological strategies toward the prevention of neurodegeneration. To address this issue, SAMP8 mice were exposed to the chronic mild stress (CMS) paradigm for 4 weeks and treated with RL-118, an 11β-hydroxysteroid dehydrogenase type 1 (11β-HSD1) inhibitor. The inhibition of this enzyme is linked with a reduction in GC levels and cognitive improvement, while CMS exposure has been associated with reduced cognitive performance. The aim of this project was to assess whether RL-118 treatment could reverse the deleterious effects of CMS on cognition and behavioral abilities and to evaluate the molecular mechanisms that compromise healthy aging in SAMP8 mice. First, we confirmed the target engagement between RL-118 and 11β-HSD1. Additionally, we showed that DNA methylation, hydroxymethylation, and histone phosphorylation were decreased by CMS induction, and increased by RL-118 treatment. In addition, CMS exposure caused the accumulation of reactive oxygen species (ROS)-induced damage and increased pro-oxidant enzymes—as well as pro-inflammatory mediators—through the NF-κB pathway and astrogliosis markers, such as GFAP. Of note, these modifications were reversed by 11β-HSD1 inhibition. Remarkably, although CMS altered mTORC1 signaling, autophagy was increased in the SAMP8 RL-118-treated mice. We also showed an increase in amyloidogenic processes and a decrease in synaptic plasticity and neuronal remodeling markers in mice under CMS, which were consequently modified by RL-118 treatment. In conclusion, 11β-HSD1 inhibition through RL-118 ameliorated the detrimental effects induced by CMS, including epigenetic and cognitive disturbances, indicating that GC-excess attenuation shows potential as a therapeutic strategy for age-related cognitive decline and AD.

## 1. Introduction

Stress is a key determinant of the healthy or pathological aging of the brain [1,2]. Stressful situations activate a neuroendocrine response, which leads to the release of catecholamines followed by glucocorticoids (GCs). Active GCs bind to their receptors, promoting slow genomic actions as well as rapid nongenomic effects, such as glucose release, lipolysis, motivation to eat palatable food, and up-regulation of the expression of anti-inflammatory cytokines [3,4]. Its release is determined by the quality, intensity, and chronicity of the stressful stimulus [5]. In the brain, stressful experiences are adaptive and necessary for the establishment of enduring memories and to facilitate the restoration of physiological and behavioral homeostasis. When stressful situations persist over a long period of time, memory formation and reasoning become impaired [6,7]. In some situations, however, the deleterious effects of stress and GCs in the brain can be transient, since stress-induced hippocampal atrophy and hippocampal-dependent behavior may be reversed after a stress-free period. However, a growing body of evidence suggests that high GC exposure in early life can adversely program the release of GCs and increase susceptibility to the development of metabolic, neuropsychiatric, and neurodegenerative diseases [8], as well as induce changes in brain structure—including the generation and loss of neurons and dendritic atrophy—and brain function, affecting electrophysiological activity and cellular signaling [9,10]. 

Several studies indicate that environmental stressors influence hypothalamic–pituitary–adrenal (HPA) axis activity and behavior by altering the methylation status of the key genes concerned with the regulation of stress responses [2,8,11]. In line with this, recent evidence has demonstrated significant associations between epigenetic alterations and stress, showing that, under the influence of chronic stressors, histone acetylation and DNA methylation are decreased, among other alterations [11,12]. Moreover, prolonged exposure to GCs is associated with immunosuppression, metabolic syndrome, diabetes, osteoporosis, reproductive failure, hypertension, and mood and affective disorders. Mal-adaptive adjustments to stress may, sequentially, lead to symptoms of depression and Alzheimer’s disease (AD). Additionally, recent clinical studies suggest that GCs are implicated in the pathogenesis and/or progression of AD [9,13]. An increase in amyloid β (Aβ) deposits and hyperphosphorylated tau have been associated with chronic stress [14,15]. Importantly, stressful events can also have long-term consequences for the immune system. Nuclear factor-κB (NF-κB) triggers a feed-forward cycle in which increased cytokine levels result in resistance to GC-induced immunosuppression, leading to further increases in cytokine release and consequent activation of the HPA axis [16,17]. Additionally, pro-inflammatory cytokines have been implicated in the genesis of AD, as well as in the formation of amyloid plaques and Aβ production. Interestingly, HPA axis activation has been linked to oxidative stress (OS) and reactive oxygen species (ROS) production [13,18,19], which are other factors involved in AD pathology and progression. Moreover, GCs are also involved in the regulation of cell fate, by modulating pro-/anti-apoptotic mechanisms and survival proteins, such as brain-derived neurotrophic factor (BDNF), inducible nitric oxide synthase (iNOS), B-cell lymphoma 2 (Bcl2), and the neural cell adhesion molecule (NCAM), suggesting that they exert significant influence over neuroplasticity [2,7,20,21]. It should be noted that the activation of autophagy becomes crucial for cell survival and longevity, as this process participates in the elimination of disrupted proteins and is implied in the maintenance of cellular homeostasis [17]. 

The localization of active GCs is controlled by the 11β-hydroxysteroid dehydrogenase type 1 (11β-HSD1) enzyme. Inhibition of this enzyme has been associated with neuroprotective effects [6,15]. In particular, we reported that RL-118, a specific 11β-HSD1 inhibitor, prevented age-related cognitive decline in mice, even under metabolic stress [15,22,23]. The aim of this study was to confirm the target engagement of RL-118 to 11β-HSD1 and to evaluate the effects of 11β-HSD1 inhibition during chronic stress exposure in a senescence mice model (SAMP8). 

## 2. Results

### 2.1. RL-118 Demonstrates Target Engagement with 11β-HSD1 Enzyme in the TAPS Assay

**Figure 1:**
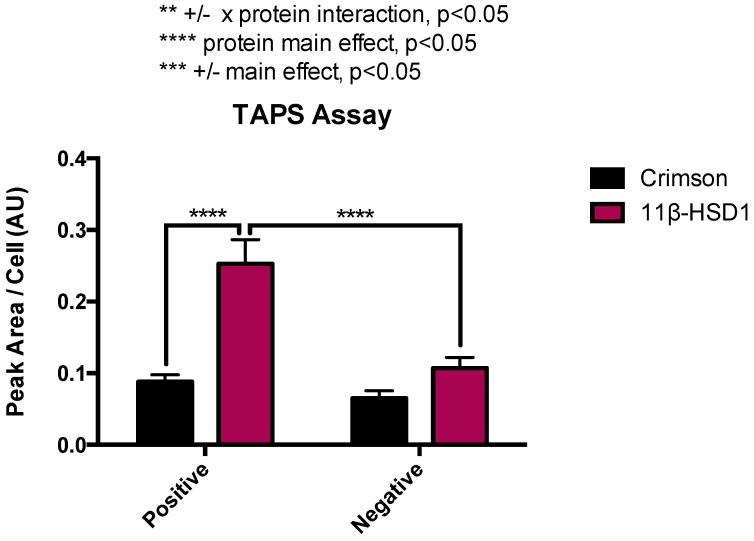
TAPS Assay results showing the peak area per cell. Values are the mean ± standard error of the mean (SEM) (*n* = 3), *** *p* < 0.0001. ** *p* < 0.01 +/− x protein interaction; *** *p* < 0.001 +/− main effect; **** *p* < 0.0001 protein main effect. The Y-axis represents the peak area obtained in LC-MS divided by the number of cells evaluated after lysis (see methodology for details).

The RL-118 target engagement was determined in the TAPS. The peak area of RL-118 quantified was relative to the number of cells and was found to be higher in cells expressing the 11β-HSD1 enzyme (11β-HSD1-positive), compared to cells found to have no expression of the enzyme following transfection (11β-HSD1-negative) (Figure 1). Additionally, the peak area was significantly higher in 11β-HSD1-positive cells than in Crimson-positive cells, indicating that the RL-118 drug is selective for 11β-HSD1. 

### 2.2. CMS-Modulated Epigenetic Marks Are Controlled by 11β-HSD1 Inhibition 

**Figure 2:**
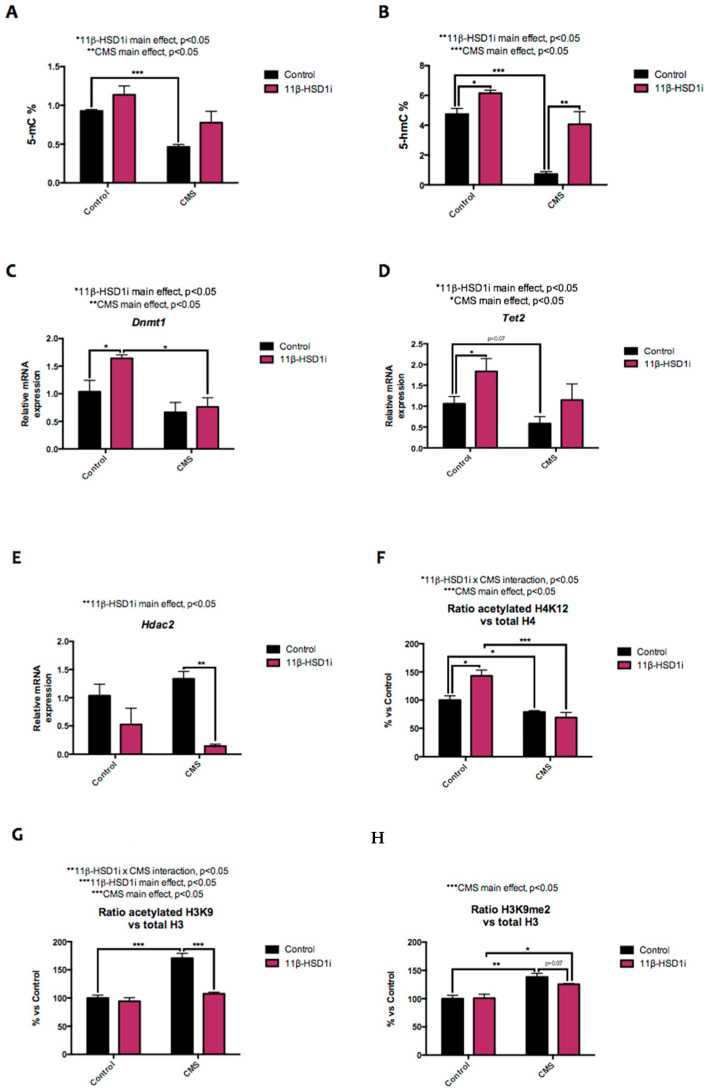
Representative results from epigenetic marks. Global 5-methylated cytosine (**A**) and 5-hydroxymethylated cytosine levels (**B**). Relative gene expression of *Dnmt1* (**C**), *Tet2* (**D**), and *Hdac2* (**E**). Representative Western blot for the ratio of Lys12 acetylated H4 protein levels and quantification (**F**), the ratio of Lys9 acetylated H3 protein levels and quantification (**G**), and the ratio of H3K9me2 protein levels and quantification (**H**). Gene expression levels were determined by real-time PCR. Western blot values in bar graphs are adjusted to 100% for protein levels of SAMP8 Control (Control). Values are mean ± standard error of the mean (SEM) (*n* = 6 for each group). 11β-HSD1 and CMS main effects as well as interactions were determined. * *p* < 0.05; ** *p* < 0.01; *** *p* < 0.001.

Regarding DNA methylation, CMS reduced global DNA methylation and 5-hydroxy methylation compared to the control group. 11β-HSD1 inhibition, meanwhile, increased DNA methylation in both 11β-HSD1 inhibitor treated groups (Figure 2A,B). In accordance with this, *DNA-methyltransferase 1 (Dnmt1)* and *ten-eleven translocase 2 (Tet2)* gene expressions were lower in the CMS-treated groups and increased in the 11β-HSD1i-treated control group (Figure 2C,D). 

As far as histone epigenetic modifications are concerned, *histone deacetylase 2 (Hdac2)* gene expression was significantly diminished by RL-118 treatment in both groups, while CMS induced a slight increase compared to the control animals (Figure 2E). The mice under CMS showed lower Lys12 acetylated histone 4 (H4K12) protein levels. In line with previous results, H4K12 protein levels were higher in the mice in the 11β-HSD1i control group (Figure 2F). Lys9 acetylated histone 3 (H3K9) and di-methylated H3K9 (H3K9me2) protein levels were higher in the CMS group and reduced after 11β-HSD1 inhibition by RL-118 (Figure 2G,H).

### 2.3. OS Increase Induced by CMS Was Prevented by RL-118 Treatment

**Figure 3:**
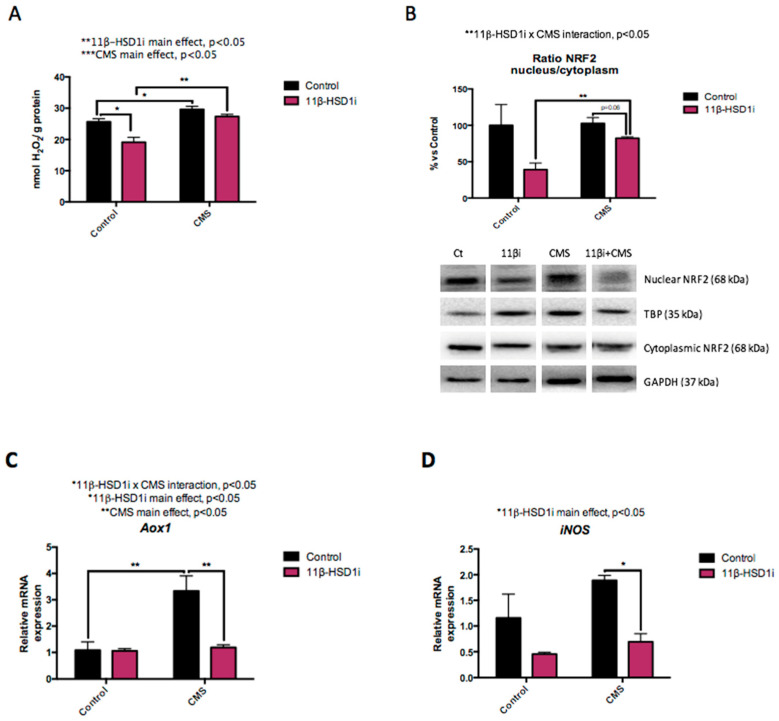
Representative results from pro-oxidant vs antioxidant mechanism imbalances. Representative ROS accumulation was measured by the hydrogen peroxide concentration in homogenates of hippocampus tissue (**A**). Representative Western blot for the ratio of nuclear/cytoplasmic NRF2 protein levels and quantification (**B**). Relative gene expression of *Aox1* (**C**) and *iNOS* (**D**). Western blot values in bar graphs are adjusted to 100% for protein levels of SAMP8 Control (Control). Gene expression levels were determined by real-time PCR. Values are mean ± standard error of the mean (SEM) (*n* = 6 for each group). 11β-HSD1 and CMS main effects as well as interactions were determined. * *p* < 0.05; ** *p* < 0.01.

CMS intensified ROS concentrations in both CMS groups compared to the control mice. However, RL-118 drug treatment contributed to a decrease in ROS levels (Figure 3A). ROS accumulation is regulated by antioxidant and pro-oxidant enzymes controlled by nuclear erythroid-related factor 2 (Nrf2), such as aldehyde oxidase 1 (Aox1). Hereby, decreased Nrf2 protein levels were observed after 11β-HSD1 inhibition, but increased levels were observed after CMS treatment (Figure 3B). In accordance, *Aox1* gene expression was significantly diminished by RL-118 in the group under CMS, but increased in the CMS control group (Figure 3C). Indeed, the same gene expression pattern was observed for *iNOS*, an enzyme involved in the synthesis of pro-oxidant molecules (Figure 3D). 

### 2.4. Pro-Inflammatory Markers Were Reduced after 11β-HSD1 Inhibition 

**Figure 4:**
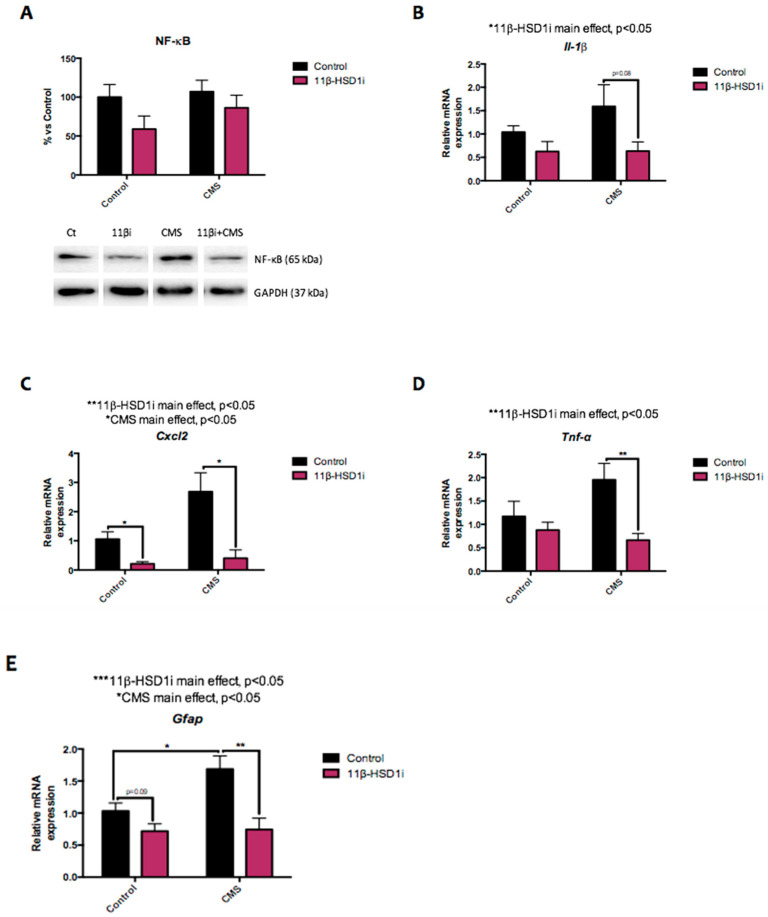
Representative results from inflammatory pathways. Representative Western blot for NF-κB protein levels and quantification (**A**). Relative gene expression of *Il-1β* (**B**), *Cxcl2* (**C**), *Tnf-α* (**D**), and *Gfap* (**E**). Western blot values in bar graphs are adjusted to 100% for protein levels of SAMP8 Control (Control). Gene expression levels were determined by real-time PCR. Values are mean ± standard error of the mean (SEM) (*n* = 6 for each group). 11β-HSD1 and CMS main effects as well as interactions were determined. * *p* < 0.05; ** *p* < 0.01.

NF-κB is a protein complex that regulates cytokine production, inflammatory signaling, and immune responses to infection. Although no statistical differences were observed in the NF-κB protein levels, the levels were decreased after RL-118 treatment in both treated groups (Figure 4A). However, there were changes in the gene expression of several cytokines controlled by this nuclear factor. CMS induced an increase in *interleukin 1β (Il-1β)*, *chemokine (C-X-C motif)*, *ligand 2 (Cxcl-2)*, and *tumor necrosis factor α (Tnf-α)* gene expressions in comparison to the control mice. By contrast, 11β-HSD1 inhibition significantly decreased cytokine gene expression in the CMS-treated mice, while in the control mice, decreased gene expression was only significant for *Cxcl-2* (Figure 4B–D). Moreover, the evaluation of *glial fibrillar acidic protein (Gfap)* gene expression demonstrated an increase due to CMS and a decrease due to 11β-HSD1 inhibition (Figure 4E).

### 2.5. 11β-HSD1 Inhibition Promoted CMS-Induced Autophagy

**Figure 5:**
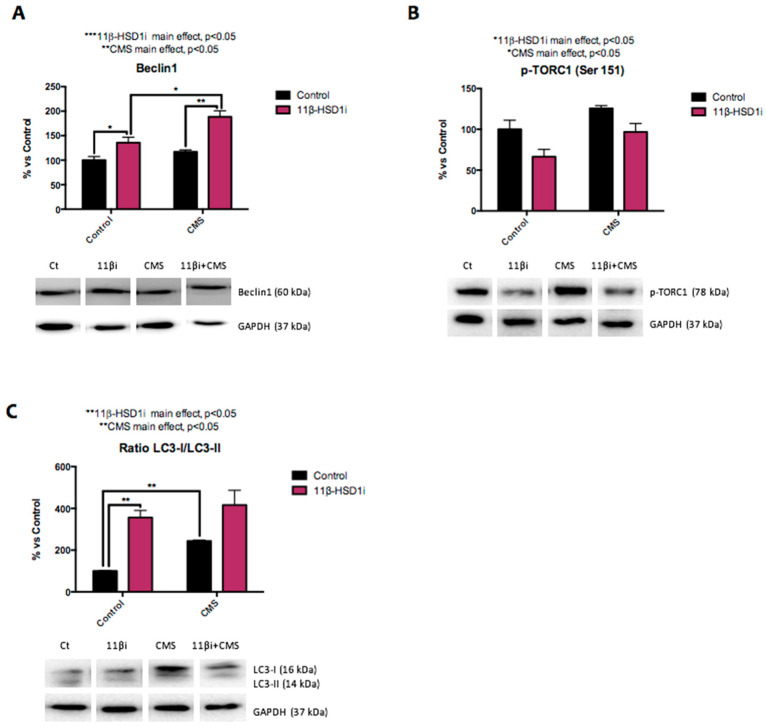
Representative results from the evaluation of the autophagy process. Representative Western blot for Beclin1 protein levels and quantification (**A**), p-TORC1 (Ser 151) protein levels and quantification (**B**), and the ratio of LC3 protein levels and quantification (**C**). Values in bar graphs are adjusted to 100% for protein levels of SAMP8 Control (Control). Values are mean ± standard error of the mean (SEM) (*n* = 4 for each group). 11β-HSD1 and CMS main effects as well as interactions were determined. * *p* < 0.05; ** *p* < 0.01.

Several autophagy markers were evaluated in this study, including Beclin1, rapamycin-sensitive TOR complex 1 (TORC1), and microtubule-associated protein 1A/1B light chain 3B (LC3B). Beclin1 and LC3B are activators of the cellular cleaning process, while TORC1 is associated with autophagy inhibition. Mice under CMS showed higher p-TORC1 (Ser151) protein levels and an elevated LC3BI/LC3II ratio (Figure 5A–C). After 11β-HSD1 inhibitor treatment, Beclin1 protein levels and the LC3BI/LC3II ratio were increased, both in the control and CMS groups, while p-TORC1 (Ser151) levels were decreased.

### 2.6. 11β-HSD1 Inhibition Rescued Mice from the Injurious Effects of CMS on APP Processing

**Figure 6:**
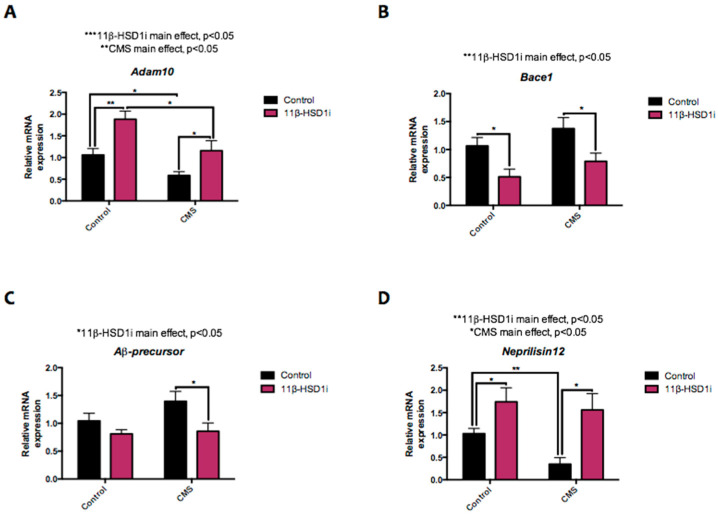
Representative results from APP processing pathways. Relative gene expression of *Adam10* (**A**), *Bace1* (**B**), *Aβ-precursor* (**C**), and *Neprilisin12* (**D**). Gene expression levels were determined by real-time PCR. Values are mean ± standard error of the mean (SEM) (*n* = 6 for each group). 11β-HSD1 and CMS main effects as well as interactions were determined. * *p* < 0.05; ** *p* < 0.01.

As shown herein, *disintegrin and metalloproteinase domain protein 10* (*Adam10*) gene expression was decreased in the CMS group and subsequently recovered by RL-118 treatment (Figure 6A). Furthermore, *β-secretase 1* (*Bace1*) gene expression was decreased in both RL-118 treated groups and slightly increased after CMS exposure (Figure 6B). In line with these results, *Aβ-precursor* gene expression was increased in the CMS group and reversed by 11β-HSD1 inhibition (Figure 6C). Finally, the β-amyloid degradation process was assessed by *Neprilisin12* gene expression. It was found that RL-118 treatment consequently increased *Neprilisin12* gene expression, which was decreased by CMS exposure (Figure 6D). 

### 2.7. 11β-HSD1 Inhibition by RL-118 Changed Synaptic Plasticity Markers after CMS 

**Figure 7:**
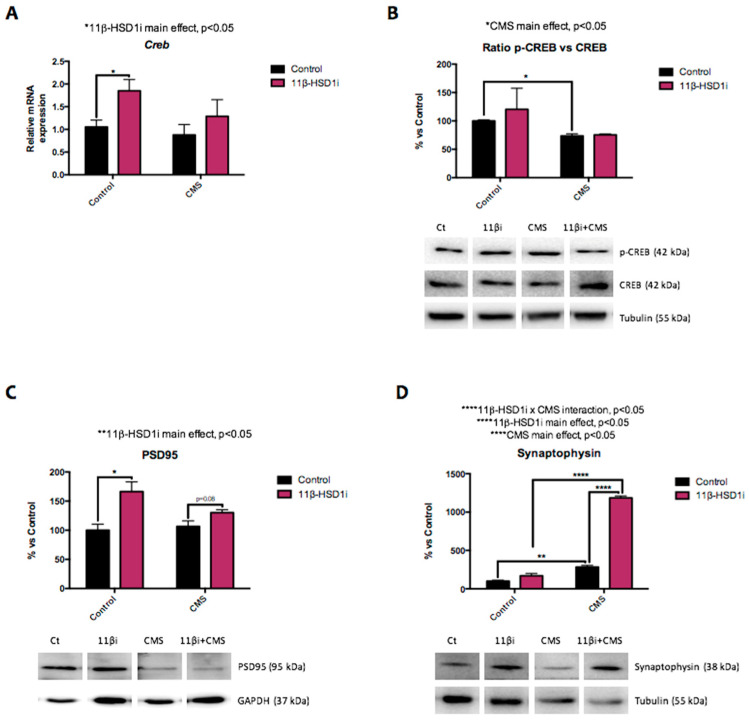
Representative results from neuroplasticity modulators. Relative gene expression of *Creb* (**A**). Representative Western blot for the ratio of p-CREB protein levels and quantification (**B**), PSD95 protein levels and quantification (**C**), and synaptophysin protein levels and quantification (**D**). Gene expression levels were determined by real-time PCR. Western blot values in bar graphs are adjusted to 100% for protein levels of SAMP8 Control (Control). Values are mean ± standard error of the mean (SEM) (*n* = 6 for each group). 11β-HSD1 and CMS main effects as well as interactions were determined. * *p* < 0.05; ** *p* < 0.01; **** *p* < 0.0001.

RL-118 increased *cAMP response element-binding (Creb)* gene expression, although this change was not significant in the CMS group. By contrast, the ratio of pCREB/CREB was reduced after CMS exposure but unmodified after RL-118 treatment (Figure 7A,B). Nevertheless, synaptic markers like postsynaptic density protein 95 (PSD95) and synaptophysin protein levels were increased after 11β-HSD1 inhibition treatment, both in the control and CMS groups (Figure 7C,D). 

### 2.8. 11β-HSD1 Inhibition Increased Memory and Learning Abilities 

**Figure 8:**
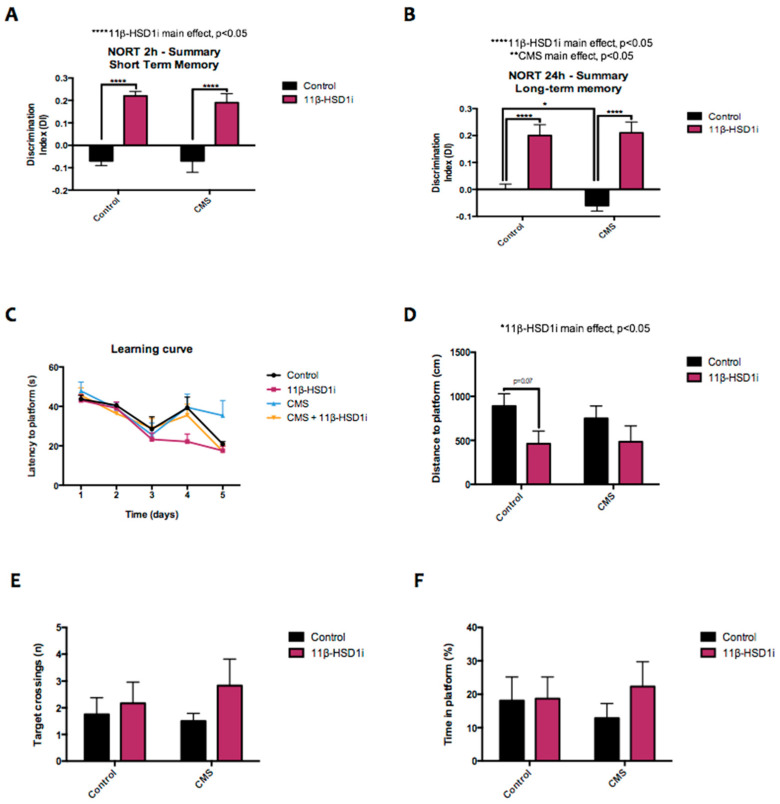
Behavioral test results from NORT and MWM respectively. Summary of DI from 2 and 24 h after familiarization phase (**A**,**B**), MWM learning curve (**C**), distance to reach the platform (**D**), number of entries (**E**), and time in the platform (**F**). Values are mean ± standard error of the mean (SEM) (*n* = 12 for each group). 11β-HSD1 and CMS main effects as well as interactions were determined. * *p* < 0.05; **** *p* < 0.0001.

We assessed recognition and spatial memory, as well as learning ability, through the evaluation of NORT and MWM. CMS did not induce significant changes in the NORT DI values for short-term memory in comparison to the control mice, although recognition memory was impaired by CMS after 24 h of familiarization (Figure 8A,B). However, 11β-HSD1-inhibitor treatment increased both short- and long-term recognition memory as the NORT DI was higher in RL-118 treated groups. On the other hand, the MWM learning curve demonstrates that all the mice learned where the platform was during the training days, as the latency to the platform was lower than the first day, although some groups performed better than others (Figure 8C). However, female mice under CMS and RL-118 treatment showed better learning ability in comparison to the CMS group, demonstrated by the higher learning curve slope in those groups. Regarding performance on the MWM test day, CMS treatment did not have a significant effect on the evaluated parameters, although a trend towards the impairment of performance was observed. By contrast, RL-118 treatment led to a subtle positive impact on mouse behavior, both in the control and CMS groups, since the distance traveled to reach the platform was reduced and was, statistically, significantly different in the control groups (Figure 8D). In line with these results, 11β-HSD1 inhibition was observed to increase target crossings as well as the time spent in the platform zone in both treated groups, compared to their littermates in the control group (Figure 8E,F).

## 3. Discussion

Impaired glucocorticoid (GC) signaling is a significant factor in aging, stress, and neurodegenerative diseases, such as Alzheimer’s disease. Therefore, the study of GC-mediated stress responses to chronic moderately stressful situations, which occur in daily life, is of huge interest for the design of pharmacological strategies toward the prevention of neurodegeneration.

Strong evidence demonstrating an association between prolonged exposure to GC excess and diminished cognitive abilities has been reported [4]. Detrimental effects induced by GCs may be the result of alterations in hippocampal electrophysiology, structure, and function, as well as the deleterious effects on neurotransmission, metabolism, and cell division and death. GC secretion responds to feed-forward signaling involving the hypothalamic–pituitary–adrenal (HPA) axis, forming the stress response. Stressful stimuli increase GC secretion and release to provide the energy necessary to cope with the stress. Mounting evidence reports that chronic stress might lead to deleterious effects on the brain, while acute stress may enhance memory [4,24]. Accordingly, it has been demonstrated that the CMS paradigm reduces cognitive abilities and increases anxiety-like behavior in mice [7,12].

Previously, we reported that RL-118, an 11β-HSD1 inhibitor, regulates the disposal of active GCs [21,22]. Importantly, 11β-HSD1 inhibition enhanced cognitive abilities and reduced AD hallmarks [15,23,25]. In accordance with these results, it has been described that 11β-HSD1 expression in a mouse’s hippocampus and parietal cortex increases with aging, and that overexpression accelerates age-related cognitive decline [26]. By contrast, 11β-HSD1 knockout mice resist age-dependent cognitive loss [27,28]. Moreover, we demonstrated that RL-118 treatment promoted autophagy flux, as well as ER stress activation, to reverse the deleterious effects exerted by prolonged exposure to GCs [15,22,23]. Overall, it is suggested that 11β-HSD1 could be a feasible target for fighting against cognitive decline in age-related pathologies. In fact, early clinical studies demonstrate that the 11β-HSD1 inhibitor (UE2343), currently in clinical phase II, is well-tolerated in AD patients [29]. Furthermore, many selective 11β-HSD1 inhibitors have reached clinical trials for metabolic diseases, for example, AZD8329 and BVT.2733 [15].

To demonstrate that RL-118 specifically binds to the 11β-HSD1 enzyme, the target engagement of RL-118 and 11β-HSD1 was determined using a novel methodology consisting of FACS (fluorescence-activated cell sorting) coupled with mass spectrometry (MS) analysis [30]. This assay of target engagement showed that RL-118 binds to its target in a selective manner since it did not bind to other proteins. Therefore, we determined that the molecular and behavioral effects observed after RL-118 administration to mice [15,22,23] were the result of binding to the 11β-HSD1 enzyme.

The stress response may have a genetic and epigenetic origin, reflected in the efficiency of the GC receptor (GR)-mediated GC negative feedback in the brain and/or the pituitary gland that causes HPA axis hyperactivity [2,11,31]. To address whether and which changes produced the chronic presence of stressors, we decided to evaluate two main epigenetic marks: DNA methylation and histone acetylation. The former is implicated in controlling neuronal gene expression and neural development. Dysregulation of this process is linked to a wide range of neuronal disorders, including AD onset and progression. However, the relationship between AD and altered 5-mC levels is not known [12,32]. The TET family of enzymes can further oxidize 5-mC to 5-hmC. Generally, 5-mC is associated with gene silencing and 5-hmC with the up-regulation of gene expressions [32,33]. According to Puigoriol-Illamola et al. [12], CMS reduces those epigenetic marks, whereas treatment with RL-118 restores the DNA methylation pattern after the detrimental stimulus. Of note, reducing GC levels by inhibiting 11β-HSD1 also increased DNA hydroxymethylation in the non-stressed mice that were treated. Likewise, the gene expressions for the enzymes responsible for these processes, *Dnmt1* and *Tet2*, were increased in both groups treated with the 11β-HSD1 inhibitor, but decreased in the CMS group, suggesting a neuroprotective effect [32,33,34]. Overall, the results suggest that further evaluation of gene-promoter-specific methylation is required to elucidate the precise epigenetic mechanisms and explain the neuroprotective effects of RL-118 that were observed in the performed cognitive tests.

DNA methylation and histone modifications must act coordinately [34]. Generally, histone acetylation is related to gene transcription by removing histone-positive charges and thereby transforming condensed chromatin (heterochromatin) into a relaxed structure (euchromatin) [35,36]. The 11β-HSD1-inhibitor treatment altered *Hdac2* gene expression, promoting a reduction in the control and CMS groups. In line with this, acetylated H4 showed higher protein levels in the RL-118-treated group, but not in the mice under CMS. In accordance with Puigoriol-Illamola et al. [12], the SAMP8 mice under CMS treatment showed higher H3K9 protein levels compared to the control group, which were decreased after RL-118 treatment. The same profile was observed in H3K9me2 protein levels, suggesting that despite the increase in histone acetylation, the increased methylation resulted in transcriptional repression or a more compacted chromatin state in the CMS groups compared to the control mice. Furthermore, although RL-118 is not a direct epigenetic-modulating drug, the reduction in GC production modified the epigenetic landscape, confirming the importance of GC-regulating epigenetics.

It is well-established that a stressful environment affects oxidative balance [12,37,38]. In normal conditions, pro-oxidant molecules and antioxidant defense mechanisms are balanced. However, in the presence of chronic stressors, as well as in aging and several disorders, there is a decrease in the capacity of antioxidant enzymes, allowing ROS accumulation. This eventually causes cellular damage and, finally, dysfunction of the system [39]. In accordance with this, the present work showed that the SAMP8 mice under CMS had an increased ROS accumulation which was prevented in the animals treated with RL-118. OS is frequently implicated as a potential factor in the progression of AD, though whether it is a cause or consequence of the pathology is still undetermined [18]. Among antioxidant defense mechanisms, the NRF2 pathway has been determined as a key indicator and modulator of OS in neurodegeneration [39,40,41], because it regulates the gene expression of different antioxidant enzymes. While NRF2 protects against oxidative and electrophilic tissue injury, persistent activation of NRF2 signaling may also contribute to disease pathophysiology [42]. Interestingly, NRF2 protein levels were decreased after RL-118 treatment, suggesting that the attenuation of GC levels induces lower oxidative damage, while CMS has the opposite effect. Accordingly, Aox1 gene expression was reduced after RL-118 treatment in the CMS group. Likewise, other OS modulators that were evaluated, such as iNOS, showed an increase in the CMS group compared to the control group and displayed decreased gene expression after RL-118 treatment. Moreover, *iNOS* gene expression has been reported to be regulated by inflammatory signaling, particularly through NF-κB [43]. Overall, results indicate that a stressful environment increases pro-oxidant mechanisms and suggest that 11β-HSD1 inhibition promotes antioxidant defenses to cope with OS in a mouse model of aging, even under CMS conditions. 

Regarding neuroinflammation, NF-κB has a pivotal role in inflammatory responses since it induces the expression of various pro-inflammatory genes, including those encoding cytokines and chemokines, and participates in inflammasome regulation [44,45]. It triggers a feed-forward cycle which increases cytokine production. This, in turn, results in resistance to GC-induced immunosuppression, leading to further increases in cytokine release and, therefore, the activation of the HPA axis [16,17]. While this result was not statistically significant, 11β-HSD1 inhibition tended to reduce NF-κB protein levels. Accordingly, we found decreased pro-inflammatory cytokine (*IL1β*, *Cxcl2*, and *TNFα*) gene expressions—which were increased due to CMS—after 11β-HSD1-inhibitor treatment. Moreover, *Il-10* and *Il-6* gene expression results reinforce this finding (data not shown). Such pro-inflammatory cytokines are known to activate the HPA axis and potentiate GC resistance [2] and have been implicated in the genesis of AD, the formation of amyloid plaques, and Aβ production, among other roles. Particularly, the dysregulation of inflammatory mediators and astrogliosis are implicated in the development of chronic inflammation and immunosenescence processes, as well as in cognitive decline and the progression of neurodegenerative diseases [45]. In agreement with this, in the current study, *Gfap* gene expression was increased in female mice under CMS and subsequently reduced after RL-118 treatment.

Recent studies have indicated that prolonged OS may limit autophagy flux [18]. In addition, altered cellular loss of proteostasis is one of the nine hallmarks of aging postulated by López-Otín et al. [46]. In accordance with previous reports, we found that GC-excess attenuation by RL-118 promoted autophagy activation in the SAMP8 mice [23], while CMS treatment induced autophagy deterioration [12]. In accordance, we report here that autophagy flux was decreased in female mice under CMS, but RL-118-treated animals showed higher protein levels of Beclin1, an increased LC3BI/II ratio, and lower protein levels of p-TORC1 Ser151 in comparison to CMS control group, indicating the recovery of autophagy in mice treated with RL-118 and under CMS. 

11β-HSD1 inhibition has been studied in association with a number of disorders, one of which is AD. It has been associated with reduced Aβ neurotoxicity and tau hyperphosphorylation [47,48], both of which are hallmarks of AD. The amyloid precursor protein (APP) can be processed through two mechanisms: non-amyloidogenic and amyloidogenic pathways. Adam10 is a marker of the former, and Bace1 is a marker of the latter [48,49]. In this study, *Adam10* gene expression was diminished and *Bace1* was increased after CMS. Importantly, the opposite was observed in RL-118 treated groups and the reported reduction in the formation of Aβ concurred with the Aβ-precursor gene expression shown here. Moreover, gene expression for the Aβ-degrading enzyme *Neprilisin12* was increased after 11β-HSD1-inhibitor treatment, but reduced after CMS. 

As mentioned, prolonged exposure to GCs in the brain induces several changes. Multiple studies state that repeated exposure to stressful conditions induces the structural remodeling of neurons with synaptic loss as well as alterations in glial functions, which are frequently maladaptive [8]. In agreement with this, our results demonstrate a reduction in p-CREB/CREB protein levels after CMS exposure, but not in *Creb* gene expression. Conversely, RL-118 treatment increased *Creb* gene expression, although it was not able to prevent the deleterious effect of CMS [50]. In a similar way, 11β–HSD1 inhibition increased synaptic plasticity markers, like PSD95 and synaptophysin. Thus, RL-118 treatment could prevent the loss of those neuroplasticity markers, particularly synaptophysin [51].

The effects of GCs on cognition have been widely studied, showing that acute stress improves cognitive abilities, while chronic stress worsens memory and learning processes and accelerates brain aging [2,4,48]. In fact, GC levels variation has been found to correlate with the severity of cognitive impairments [48]. In accordance with Puigoriol-Illamola et al. [12,15], the mice under RL-118 treatment clearly showed improved recognition memory when compared with the control mice. However, the injurious effects caused by CMS were only detected during the evaluation of long-term recognition memory. Slight changes were also observed in spatial memory, where 11β-HSD1 inhibition was observed to improve the performance of mice, although a statistical difference was not detected. However, RL-118 induced improved learning abilities in the CMS group, as the learning curve slope was higher for the treated groups, indicating a putative role for RL-118 as a neuroprotectant or cognition-enhancer.

It should be noted that one of the limitations of this study was that CMS was applied to a strain of senescent mice with high basal levels of stress, inflammation, and OS, as reported in Puigoriol et al., 2020 [12]. Therefore, it is possible that the CMS treatment did not worsen cognitive abilities or certain stress markers due to the specific aging characteristics of the SAMP8 mice. In fact, the beneficial effects obtained by the inhibition of 11β-HSD1 by RL-118 in the mice under CMS emphasize the importance of controlling GC levels to mitigate the harmful effects of aging and stressful lifestyles.

**Figure 9:**
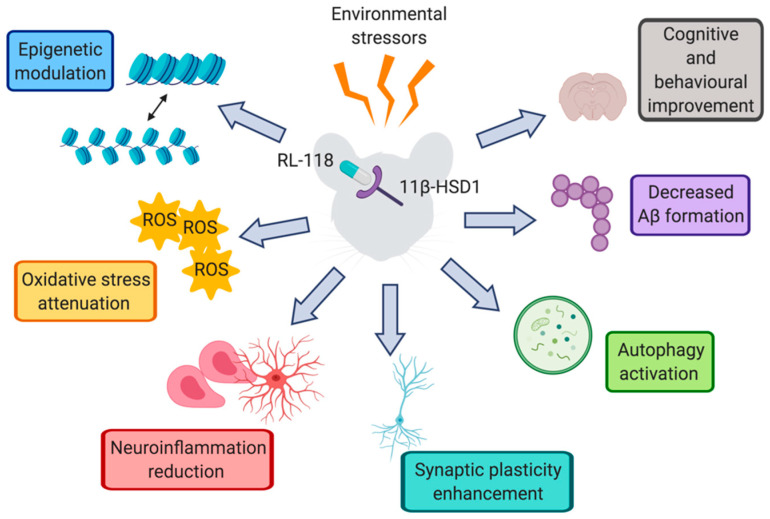
Representative scheme of the molecular pathways altered after RL-118 treatment in SAMP8 mice under chronic mild stress. Authors should discuss the results and how they can be interpreted from the perspective of previous studies and of the working hypotheses. The findings and their implications should be discussed in the broadest context possible. Future research directions may also be highlighted.

This study demonstrates the target engagement between RL-118 and the 11β-HSD1 enzyme. Therefore, we can attribute the beneficial effects observed in the SAMP8 mice treated with RL-118 to 11β-HSD1 inhibition. 11β-HSD1 inhibition by RL-118 was found to improve most of the detrimental effects of CMS—including the enhancement of cognitive decline and antioxidant mechanisms—as well as improving synaptic plasticity and autophagy markers. Additionally, the RL-118 treatment modulated epigenetic markers and reduced inflammatory signaling and Aβ formation and accumulation (Figure 9). 

## 4. Materials and Methods

### 4.1. Cloning

The human E2-Crimson HSD11B1 gene (variant 1) was synthesized by GenScript in vector pUC57. The DNA sequence for E2-Crimson was sourced from Clontech. The gene for the fluorescent-HSD11B1 was ligated into vector pcDNA3.1 (Invitrogen) using restriction sites NheI (N-terminus) and NotI (C-terminus) (Figure S1). 

### 4.2. Transient Expression of the Fluorescent Target Protein 

HEK293 cells were passaged in poly-D-Lysine treated plates and incubated overnight in OPTI-MEM medium (Lonza) at 37 °C, 5% CO_2_. The cells were transiently transfected the following day with pcDNA3.1-E1-Crimson-huHSD11B1 DNA using Lipofectamine 2000 (Invitrogen) in OPTI-MEM medium via standard transfection protocol. The transfected cells were maintained at 37 °C, 5% CO_2_ for 24 h post-transfection before the TAPS assay was performed. Transfection of the cells and compound screening were performed in a 6-well plate format. 

### 4.3. TAPS Assay

#### 4.3.1. Compound Incubation

The RL-118 drug was diluted to a concentration of 20 μM (DMSO < 1%) in a tissue culture medium (DMEM with 10% FBS, 1% L-Glutamine, 1% penicillin-streptomycin, (Life Technologies, Carlsbad, CA, USA)), then incubated with transfected cells for three hours at 37 °C, 5% CO_2_. A 20 μM compound concentration was selected as an appropriate concentration for assay development and screening. Following incubation, the cells were detached from the plate by gentle pipetting and centrifuged for 5 min at 1000 rpm. The culture medium was removed by pipetting and the cell pellet was re-suspended in FACS buffer (PBS + 2% FBS) to wash off unbound compounds. The cells were centrifuged as above, and the wash buffer was removed. The cells were then resuspended in FACS buffer and transferred to 5 mL FACS tubes. The tubes were wrapped in foil and placed on ice until sorting. 

#### 4.3.2. FACS Sorting 

Cells were sorted using a BD FACS Aria II system fitted with a 100 μm nozzle. Data were acquired and processed using BD FACS Diva software version 8.0.1. Cell fluorescence was detected using a 640 nm laser for E2-Crimson excitation. The filter used was 670/14 nm, detecting fluorescence emission of E2-Crimson in the 663–677 nm range. Cells were sorted and collected into four cell populations defined by fluorescence intensity. Forward- (FSC) and side-scatter (SSC) gating was used to exclude dead cells and only live cells were collected. Cells were collected in 5 mL FACS tubes containing 1 mL of DMEM with 10% FBS, 1% L-Glutamine, and 1% penicillin-streptomycin. Each population of cells was centrifuged at 1000 rpm for 5 min. The medium was removed, and the cell pellet was resuspended in 20 mM of HEPES, pH 7.0. The suspension was briefly sonicated to lyse the cells before centrifugation, as before, to pellet cell debris. The lysate (supernatant) was transferred to LC-MS vials and stored at −20°C prior to MS analysis. 

#### 4.3.3. Mass Spectrometry Detection of the Compounds in Cell Lysates

The chromatographic and mass spectrometer used was the SCIEX Triple Quad 5500+ LC-MS/MS System- QTRAP (Triple Quad^TM^, Macclesfield, UK). A 10 μL injection of the cell lysate was loaded directly onto an HSST3 (150 mm × 2.1 mm, Thermo Fisher Scientific, Waltham, MA, USA) column at a high flow rate, causing the proteinaceous material to flow to waste. A series of valve switches led to the elution of the extracted sample from the column directly onto the analytical column. Solvent A was water with 0.1% formic acid and solvent B was methanol with 0.1% formic acid. Automated tune settings were used to achieve the maximum ion signal for the analyte for initial validation experiments, optimizing tube lens voltage, parent-to-product transitions, and collision energy for transition. Peaks detected in this initial scan were then identified by molecular weight and checked versus their mass-charge ratio. Data were acquired and processed using Sciex OS-MQ software (Macclesfield, UK).

### 4.4. Animals

Four-month-old female SAMP8 mice (*n* = 48) were used to carry out behavioral, cognitive, and molecular analyses. We divided these animals into four groups: SAMP8 Control (Control, *n* = 12), SAMP8 treated with RL-118 (11β-HSD1i, *n* = 12), SAMP8 under CMS (CMS, *n* = 12), and SAMP8 treated with RL-118 under CMS (SAMP8 11β-HSD1i + CMS, *n* = 12). Animals had free access to food and water and were kept under standard temperature conditions (22 ± 2 °C) and 12 h:12 h light–dark cycles (300 lux/0 lux). RL-118 was administered at 21 mg/kg/day by oral gavage for 4 weeks. The dose administered was in accordance with previous works [15,22,23], which demonstrated the efficacy of 21 mg/kg of RL-118 for 4 weeks in mice. The chronic stressful stimuli consisted of several different stressful stimuli applied to the corresponding animals daily for 4 weeks and was called chronic mild stress (CMS) treatment. 

Studies and procedures involving mice brain dissection and subcellular fractionation were performed following standard ethical guidelines (European Communities Council Directive 86/609/EEC) and were approved by the Institutional Animal Care and Use Committee of the University of Barcelona (670/14/8102) and by Generalitat de Catalunya (10291). 

### 4.5. Chronic Mild Stress Treatment

The CMS procedure used in the present study has been previously validated in SAMR1 and SAMP8 mice [12]. For 4 weeks, mice were exposed daily to various randomly scheduled, low-intensity environmental stressors. The number and the type of stressful events executed changed every day, as well as the sequence of the stressors, to guarantee the degree of unpredictability [12]. Stressful stimuli included 2 h of physical restraint, 24 h of sawdust removal, 24 h of food deprivation, 24 h of water deprivation, 24 h of wet bedding, 1 min of tail nipping at 1 cm from the tip of the tail, and overnight illumination. 

### 4.6. Behavioral and Cognitive Tests

#### 4.6.1. Novel Object Recognition Test (NORT)

The Novel Object Recognition Test (NORT) protocol employed was as described in Puigoriol-Illamola et al. [15]. In brief, mice were placed in a 90°, two-arms, 25-cm-long, 20-cm-high, 5-cm-wide black maze. Before performing the test, the mice were individually habituated to the apparatus for 10 min for 3 days. On day 4, the animals were submitted to a 10-min acquisition trial (first trial), during which they were placed in the maze in the presence of two identical, novel objects at the end of each arm. After a delay (2 h and 24 h), the animal was exposed to two objects: one old object and one novel object. The time that mice explored the novel object (TN) and the time that mice explored the old object (TO) were measured. A discrimination index (DI) was defined as (TN − TO)/(TN + TO). To avoid object preference biases, objects were counterbalanced. The maze, the surface, and the objects were cleaned with 70% ethanol between the animals’ trials to eliminate olfactory cues. 

#### 4.6.2. Morris Water Maze (MWM)

This test evaluates both learning and spatial memory [52,53]. An open, circular pool (100 cm in diameter, 50 cm in height) filled with water was used. The water was painted white with latex to make it opaque, and its temperature was 22 ± 1 °C. Two main perpendicular axes were established (north–south and east–west), thus configuring four equal quadrants (NE, NW, SE, and SW). Four visual clues (N, S, E, W) were placed on the walls of the tank so that the animal could orientate and fulfill the objective. The test consists of training a mouse to find a submerged platform (learning phase) and assesses whether the animal has learned, so that it could remember the platform’s location after its removal (test). The training lasts five consecutive days and every day five trials are performed—using different starting points (NE, E, SE, S, and SW)—with the aim that the animal recognizes the visual clues and learns how to locate the platform, avoiding learning a static path. For each trial, the mouse was placed gently into the water, facing the wall of the pool, and allowed to swim for 60 s. There was no resting time between trials. If the animal was not able to locate the platform, the investigator guided it to the platform and allowed it to rest and orientate for 30 s. The platform was placed approximately in the middle of one of the quadrants, 1.5 cm below the water level. Above the pool, there was a camera that recorded the animals’ swimming paths, and the data were analyzed with the statistical program SMART^®^ ver.3.0 (Panlab, Barcelona, Spain). During the learning phase, a learning curve was drawn, representing the latency to find the platform on each training day. On the test day, the platform was removed, and more parameters were measured, such as the target crossings and the distance swum to reach the platform zone.

### 4.7. Brain Processing

Three days after the behavioral and cognitive tests, 12 animals per group were euthanized for protein extraction and RNA and DNA isolation. The brains were immediately removed from the skull and the hippocampi were isolated, frozen on powdered dry ice, and maintained at −80 °C until the procedures. 

### 4.8. Western Blotting

Tissue samples were homogenized in lysis buffer (Tris HCl pH 7.4 50 mM, NaCl 150 mM, EDTA 5 mM, and 1 X-Triton X-100) containing phosphatase and protease inhibitors (Cocktail II, Sigma-Aldrich, St. Louis, MI, USA) to obtain total protein homogenates. For subcellular fractionation, 150 μL of buffer A (10 mM HEPES pH 7.9, 10 mM KCl, 0.1 mM EDTA pH 8, 0.1 mM EGTA pH 8, 1 mM DTT, 1 mM PMSF, protease inhibitors) were added to each sample and incubated on ice for 15 min. After this time, the samples were homogenized with a tissue homogenizer, and 12.5 μL of Igepal 1% was added and mixed for 15 s. Following 30 s of full-speed centrifugation at 4 °C, supernatants were collected (cytoplasmic fraction); 80 μL of buffer C (20 mM HEPES pH 7.9, 0.4 M NaCl, 1 mM EDTA pH 8, 0.1 mM EGTA pH 8, 20% Glycerol 1 mM DTT, 1 mM PMSF, protease inhibitors) were added to each pellet and incubated under agitation at 4 °C for 15 min. Subsequently, samples were centrifuged for 10 min at full speed at 4 °C. Supernatants were collected (nuclear fraction) and 40 μL of buffer A + HCl (buffer A with 0.2 N HCl) were added to the pellet. After a 30 min incubation on ice, samples were centrifuged, again at full speed, at 4 °C for 10 min and the supernatants were collected (the histone fraction). Aliquots of 15 μg of hippocampal protein extraction per sample were used. Protein samples were separated by sodium dodecyl sulphate-polyacrylamide gel electrophoresis (SDS-PAGE) (8–14%) and transferred onto polyvinylidene difluoride (PVDF) membranes (Millipore). Afterward, the membranes were blocked in 5% non-fat milk in Tris-buffered saline (TBS) solution containing 0.1% Tween 20 TBS (TBS-T) for 1 h at room temperature, followed by overnight incubation at 4 °C with the primary antibodies listed in (Table S1). Then, the membranes were washed and incubated with the secondary antibodies listed in (Table S1) for 1 h at room temperature. Immunoreactive proteins were viewed with the chemiluminescence-based ChemiLucent^TM^ detection kit, following the manufacturer’s protocol (ECL Kit, Millipore), and digital images were acquired using the ChemiDoc XRS + System (BioRad). Semi-quantitative analyses were done using ImageLab software (BioRad) and results were expressed in Arbitrary Units (AU), considering control protein levels as 100%. Protein loading was routinely monitored by immunodetection of glyceraldehyde-3-phosphate dehydrogenase (GAPDH), β-tubulin, or TATA-Binding protein (TBP). 

### 4.9. RNA Extraction and Gene Expression Determination by q-PCR

Total RNA isolation was carried out using TRIsureTM reagent according to the manufacturer’s instructions (Bioline Reagent, UK). The yield, purity, and quality of the RNA were determined spectrophotometrically with a NanoDrop™ ND-1000 (Thermo Scientific) apparatus and an Agilent 2100B Bioanalyzer (Agilent Technologies, Santa Clara, CA, USA). RNAs with 260/280 ratios and RIN higher than 1.9 and 7.5, respectively, were selected. A reverse transcription-polymerase chain reaction (RT-PCR) was performed as follows: 2 μg of messenger RNA (mRNA) was reverse-transcribed using the High-Capacity cDNA Reverse Transcription Kit (Applied Biosystems, Waltham, MA, USA). Real-time quantitative PCR (qPCR) was used to quantify mRNA expressions of oxidative stress and inflammatory genes listed in (Table S2). SYBR^®^ Green real-time PCR was performed in a Step One Plus Detection System (Applied-Biosystems, Waltham, MA, USA) employing SYBR^®^ Green PCR Master Mix (Applied-Biosystems, Waltham, MA, USA). Each reaction mixture contained 6.75 μL of complementary DNA (cDNA) (of which concentration was 2 μg), 0.75 μL of each primer (of which concentration was 100 nM), and 6.75 μL of SYBR^®^ Green PCR Master Mix (2×). 

Data were analyzed utilizing the comparative cycle threshold (Ct) method (ΔΔCt), where the housekeeping gene level was used to normalize differences in sample loading and preparation. The normalization of expression levels was performed with β-actin for SYBR^®^ Green-based real-time PCR results. Each sample was analyzed in duplicate, and the results represent the n-fold difference of the transcript levels among the different groups.

### 4.10. Global DNA Methylation and Hydroxymethylation Determination 

The isolation of genomic DNA was conducted using the FitAmp^TM^ Blood and Cultured Cell DNA Extraction Kit (EpiGentek, Farmingdale, NY, USA) according to the manufacturer’s instructions. Following this, the Methylflash Methylated DNA Quantification Kit (Epigentek, Farmingdale, NY, USA) and the MethylFlash HydroxyMethylated DNA Quantification Kit were used in order to detect methylated and hydroxymethylated DNA. In brief, these kits are based on the specific antibody detection of 5-mC and 5-hmC residues, which trigger an ELISA-like reaction that allows colorimetric quantification of global DNA methylation and 5-hydroxymethylation at 450 nm. Both kits were used following the manufacturer’s instructions, including internal standards for methylated and hydroxymethylated DNA.

### 4.11. Oxidative Stress Determination 

Hydrogen peroxide was measured in hippocampus protein homogenates as an indicator of oxidative stress and was quantified using the Hydrogen Peroxide Assay Kit (Sigma-Aldrich, St. Louis, MI, USA) according to the manufacturer’s instructions. In brief, the kit is based on detecting a red fluorescent product (λex = 540/λem = 590 nm) generated after a reaction with hydrogen peroxide and can be analyzed by a fluorescent microplate reader.

### 4.12. Data Analysis

The data analysis was conducted using GraphPad Prism ver. 7 statistical software. Data are expressed as the mean ± standard error of the mean (SEM) of at least 6 samples per group and 3 different experiments for the TAPS assay. CMS and drug treatment effects were assessed by the Two-Way ANOVA analysis of variance, followed by a Tukey post-hoc analysis or a two-tail Student’s t-test where necessary. Statistical significance was considered when *p*-values were <0.05. The main effects and significant interaction are shown in each graph. The statistical outliers were determined with Grubbs’ test and subsequently removed from the analysis.

## 5. Conclusions

In conclusion, 11β-HSD1 inhibition through RL-118 treatment ameliorated the detrimental effects induced by CMS, including epigenetic and cognitive disturbances. This indicates that GC-excess attenuation has potential as a therapeutic strategy for age-related cognitive decline and AD.

## Data Availability

Data is contained within the article and Supplementary Materials.

## Supplementary Materials

The following are available online at https://www.mdpi.com/article/10.3390/ph14101040/s1, Figure S1: HSD11B1 Protein sequence, HSD11B1 DNA sequence, and Fusion Protein design; Table S1: Antibodies used in Western blot studies; Table S2: Primers and probes used in qPCR studies.
