# Supplementary material for: Inhibition of 11β-HSD1 Ameliorates Cognition and Molecular Detrimental Changes after Chronic Mild Stress in SAMP8 Mice
Source: Pharmaceuticals (Basel). 2021 Oct 13;14(10):1040. doi: 10.3390/ph14101040 (PMC8540242; doi:10.3390/ph14101040)
Supplement: Supplementary file 1 [file pharmaceuticals-14-01040-s001.zip › pharmaceuticals-1391063-supplementary.pdf]

Figure S1

Protein sequence

```
ORIGIN
1 mafmkkyllp ilglfmayyy ysaneefrpe mlqgkkvivt gaskgigrem ayhlakmgah
61 vvvtarsket lqkvvshcle lgaasahyia gtmedmtfae qfvaqagklm ggldmlilnh
121 itntslnlfh ddihhvrksm evnflsyvvl tvaalpmlkq sngsivvvss lagkvaypmv
181 aaysaskfal dgffssirke ysvsrvnvsi tlcvlglidt etamkavsgi vhmqaapkee
241 caleiikgga lrqeevyyds slwttllirn pcrkilefly stsynmdrfi nk
```

HSD11B1 DNA sequence

```
ORIGIN
1    acaattcaga ggctgctgcc tgcttaggag gttgtagaaa gctctgtagg ttctctctgt
61   gtgtcctaca ggagtcttca ggccagctcc ctgtcggatg gcttttatga aaaaatatct
121  cctccccatt ctggggctct tcatggccta ctactactat tctgcaaacg aggaattcag
181  accagagatg ctccaaggaa agaaagtgat tgtcacaggg gccagcaaag ggatcggaag
241  agagatggct tatcatctgg cgaagatggg agcccatgtg gtggtgacag cgaggtcaaa
301  agaaactcta cagaaggtgg tatcccactg cctggagctt ggagcagcct cagcacacta
361  cattgctggc accatggaag acatgacctt cgcagagcaa tttgttgccc aagcaggaaa
421  gctcatggga ggactagaca tgctcattct caaccacatc accaacactt ctttgaatct
481  ttttcatgat gatattcacc atgtgcgcaa aagcatggaa gtcaacttcc tcagttacgt
541  ggtcctgact gtagctgcct tgcccatgct gaagcagagc aatggaagca ttgttgtcgt
601  ctcctctctg gctgggaaag tggcttatcc aatggttgct gcctattctg caagcaagtt
661  tgctttggat gggttcttct cctccatcag aaaggaatat tcagtgtcca gggtcaatgt
721  atcaatcact ctctgtgttc ttggcctcat agacacagaa acagccatga aggcagtttc
781  tgggatagtc catatgcaag cagctccaaa ggaggaatgt gccctggaga tcatcaaagg
841  gggagctctg cgccaagaag aagtgtatta tgacagctca ctctggacca ctcttctgat
901  cagaaatcca tgcaggaaga tcctggaatt tctctactca acgagctata atatggacag
961  attcataaac aagtaggaac tccctgaggg ctgggcatgc tgagggattt tgggactgtt
1021 ctgtctcatg tttatctgag ctcttatcta tgaagacatc ttcccagagt gtccccagag
1081 acatgcaagt catgggtcac acctgacaaa tggaaggagt tcctctaaca tttgcaaaat
1141 ggaaatgtaa taataatgaa tgtcatgcac cgctgcagcc agcagttgta aaattgttag
1201 taaacatagg tataattacc agatagttat attaaattta tatcttatat ataataatat
1261 gtgatgatta atacaatatt aattataata aaggtcacat aaactttata aattcataac
1321 tggtagctat aacttgagct tattcaggat ggtttcttta aaaccataaa ctgtacaaat
1381 gaaa
```

Fusion Protein design

NheI Kozak

5’GCTAGC**GCCACCATGGCG**AGC**ATG**GATAGCACTGAGAACGTCATCAAGCCCTTCATGCGCTTCAAGGT
GCACATGGAGGGCTCCGTGAACGGCCACGAGTTCGAGATCGAGGGCGTGGGCGAGGGCAAGCCCTACGAG
GGCACCCAGACCGCCAAGCTGCAAGTGACCAAGGGCGGCCCCCTGCCCTTCGCCTGGGACATCCTGTCCC
CCCAGTTCTTCTACGGCTCCAAGGCGTACATCAAGCACCCCGCCGACATCCCCGACTACCTCAAGCAGTC
CTTCCCCGAGGGCTTCAAGTGGGAGCGCGTGATGAACTTCGAGGACGGCGGCGTGGTGACCGTGACCCAG
GACTCCTCCCTGCAGGACGGCACCCTCATCTACCACGTGAAGTTCATCGGCGTGAACTTCCCCTCCGACG
GCCCCGTAATGCAGAAGAAGACTCTGGGCTGGGAGCCCTCCACTGAGCGCAACTACCCCCGCGACGGCGT
GCTGAAGGGCGAGAACCACATGGCGCTGAAGCTGAAGGGCGGCGGCCACTACCTGTGTGAGTTCAAGTCC
ATCTACATGGCCAAGAAGCCCGTGAAGCTGCCCGGCTACCACTACGTGGACTACAAGCTCGACATCACCT
CCCACAACGAGGACTACACCGTGGTGGAGCAGTACGAGCGCGCCGAGGCCCGCCACCACCTGTTCCAGGA
TGACGATGACAAG**ATG**GCTTTTATGAAAAAATATCTCCTCCCCATTCTGGGGCTCTTCATGGCCTACTAC
TACTATTCTGCAAACGAGGAATTCAGACCAGAGATGCTCCAAGGAAAGAAAGTGATTGTCACAGGGGCCA
GCAAAGGGATCGGAAGAGAGATGGCTTATCATCTGGCGAAGATGGGAGCCCATGTGGTGGTGACAGCGAG
GTCAAAAGAAACTCTACAGAAGGTGGTATCCCACTGCCTGGAGCTTGGAGCAGCCTCAGCACACTACATT
GCTGGCACCATGGAAGACATGACCTTCGCAGAGCAATTTGTTGCCCAAGCAGGAAAGCTCATGGGAGGAC
TAGACATGCTCATTCTCAACCACATCACCAACACTTCTTTGAATCTTTTTCATGATGATATTCACCATGT
GCGCAAAAGCATGGAAGTCAACTTCCTCAGTTACGTGGTCCTGACTGTAGCTGCCTTGCCCATGCTGAAG

CAGAGCAATGGAAGCATTGTTGTCGTCTCCTCTCTGGCTGGGAAAGTGGCTTATCCAATGGTTGCTGCCT
ATTCTGCAAGCAAGTTTGCTTTGGATGGGTTCTTCTCCTCCATCAGAAAGGAATATTCAGTGTCCAGGGT
CAATGTATCAATCACTCTCTGTGTTCTTGGCCTCATAGACACAGAAACAGCCATGAAGGCAGTTTCTGGG
ATAGTCCATATGCAAGCAGCTCCAAAGGAGGAATGTGCCCTGGAGATCATCAAAGGGGGAGCTCTGCGCC
AAGAAGAAGTGTATTATGACAGCTCACTCTGGACCACTCTTCTGATCAGAAATCCATGCAGGAAGATCCT
GGAATTTCTCTACTCAACGAGCTATAATATGGACAGATTCATAAACAAG**TAG**GCGGCCGC**3’**
Not1

Table S1. Antibodies used in Western blot studies.

Antibody	Host	Source/Catalog	WB dilution
**Acetyl H4K12**	Sheep	R&D Systems/AF5215	1:1000
**H4**	Rabbit	Cell Signaling/#2592	1:1000
**Acetyl H3K9**	Rabbit	Millipore/06-599	1:1000
**H3**	Rabbit	Cell Signaling/#9715	1:1000
**H3K9me2**	Rabbit	Cell Signaling/#4658	1:1000
**NF-κβ**	Rabbit	Cell Signaling/DE14E12	1:1000
**NRF2**	Rabbit	Santa Cruz/sc-722	1:500
**Beclin 1**	Rabbit	Abcam/ab62557	1:1000
**p-TORC1 S151**	Rabbit	Cell Signaling/#3359	1:1000
**TORC1**	Rabbit	Cell Signaling/#2501	1:1000
**LC3B**	Rabbit	Cell Signaling/#2775	1:1000
**p-CREB S133**	Rabbit	Cell Signaling/#9198	1:1000
**CREB**	Rabbit	Cell Signaling/#4820	1:1000
**BDNF**	Rabbit	Santa Cruz/N-20	1:500
**PSD95**	Rabbit	Abcam/ab12093	1:2000
**Synaptophysin**	Mouse	Millipore/MAB5258	1:1000
**SNAP25**	Mouse	Santa Cruz/ SP-12	1:500
**GAPDH**	Mouse	Millipore/MAB374	1:2000
**β-Tubulin**	Mouse	Millipore/Clone AA2	1:2000
**TBP**	Mouse	Abcam/ab818	1:2000
**Goat-anti-mouse HRP conjugated**		Biorad/170-5047	1:2000
**Goat-anti-rabbit HRP conjugated**		Biorad/170-6515	1:2000
**Donkey-anti-goat HRP conjugated**		Santa Cruz/sc-2020	1:2000

Table S2. Primers and probes used in qPCR studies.

SYBR-Green primers

Target	Product size (bp)	Forward primer (5'-3')	Reverse primer (5'-3')
Tet2	113	CCATCATGTTGTGGGACGGA	ATTCTGAGAACAGCGACGGT
Hdac2	280	CTATCCCGCTCTGTGCCCT	GAGGCTTCATGGGATGACCC
Il-1β	179	ACAGAATATCAACCAACAAGTTG ATATTCTC	GATTCTTTCCTTTGAGGCCCA
Cxcl2	100	AGCCACACTTCAGCCTAGCG	TGTAGCCTGGTGGTTGGTGG
Tnf-α	157	TCGGGGTGATCGGTCCCCAA	TGGTTTGCTACGACGTGGGCT
Gfap	125	CCTTCTGACACGGATTTGGT	ACATCGAGATCGCCACCTAC
Aox1	286	CATAGGCGGCCAGGAACATT	TCCTCGTTCCAGAATGCAGC
iNOS	101	GGCAGCCTGTGAGACCTTTG	GAAGCGTTTCGGGATCTGAA
Adam10	125	CCTTCTGACACGGATTTGGT	CTGTACAGCAGGGTCCTTGAC
Bace1	67	ACAAGCCTTTCCGCCTCC	TCAGGCCACCATAATCCAGC
Aβ-precursor	99	TCGGGGTGATCGGTCCCCAA	GTCACGTTCACCCTCCCCAG
Neprilisin12	196	TTGGGAGACCTGGCGGAAAC	CATTCCTTGGACCCTCACCCC
Creb	86	GGCTGCTGCTGCCTGT	ACACACCGCGTCAAACTACA
β-actin	218	CTGTCCCTGTATGCCTCTG	ATGTCACGCACGATTTCC

Taqman probes

Target	Product size (bp)	Reference
Dnmt1	58	Mm01151063_m1
Gapdh	107	Mm99999915_g1
